# Supplementary material for: Integrative proteogenomic analysis identifies COL6A3-derived endotrophin as a mediator of the effect of obesity on coronary artery disease
Source: Nat Genet. 2025 Jan 24;57(2):345–57. doi: 10.1038/s41588-024-02052-7 (PMC11821532; doi:10.1038/s41588-024-02052-7)
Supplement: Supplementary file 1 — Supplementary Notes 1–9 and Figs. 1–4. [file 41588_2024_2052_MOESM1_ESM.pdf]

nature genetics

Supplementary information https://doi.org/10.1038/s41588-024-02052-7

Integrative proteogenomic analysis identifies COL6A3-derived endotrophin as a mediator of the effect of obesity on coronary artery disease

In the format provided by the authors and unedited

Supplementary Information

Table of contents:

Supplementary Note 1: Potential bias due to sample overlap in two-sample MR.
Supplementary Note 2: Simulation analysis to evaluate the false positive control of two-step approach.
Supplementary Note 3: The effect of rs11677932 on Subcutaneous Adipose-Derived Mesenchymal Cells (AMSC).
Supplementary Note 4: Discussion on PCSK9 and F11.
Supplementary Note 5: Challenges regarding the estimation of the proportion mediated in MR mediation analysis with *cis*-pQTL.
Supplementary Note 6: STROBE-MR checklist of recommended items to address in reports of Mendelian randomization studies.
Supplementary Note 7: Definition of coronary artery disease in the EPIC-Norfolk analysis.
Supplementary Note 8: GWAS of the sex-stratified pQTL for C-terminal COL6A3**.**
Supplementary Note 9: Quality control in the single-cell RNA sequencing analysis.

Supplementary Note 1: Potential bias due to sample overlap in two-sample MR.

Additionally, we evaluated the potential bias due to sample overlap in two-sample MR in both Step 1 and Step 2. Sample overlap between the exposure and outcome GWAS can bias the causal estimate either towards the null or in the direction of the observational association between the risk factor and outcome. Relative bias, which quantifies the extent to which MR's causal estimate is biased due to sample overlap relative to the observational estimate[1], is calculated as:

$$Relative\ bias = \phi \times \frac{1}{F}$$

where ϕ is the proportion of the sample overlap (ranging from 0 to 1) and F is the F-statistic of the exposure.
In Step 1, we estimated the causal effect of BMI on protein levels. The F-statistic for BMI was 94.16, and the sample overlap was at most 3.93%, given that the deCODE cohort contributed data for 26,799 individuals to the GIANT consortium, and the GWAS of BMI included 693,529 individuals. Thus, the maximum relative bias for Step 1 MR was calculated to be 0.0418% (see **Methods** and **Supplementary Table 10**).

In Step 2 MR, we estimated the causal effects of protein levels on coronary artery disease, ischemic stroke, cardioembolic stroke, and type 2 diabetes. The median F-statistic for protein levels was 272.42 (25th percentile: 103.24, 75th percentile: 660.48, minimum: 27.48) (**Supplementary Table 2**). The maximum potential sample overlap between the outcome GWAS and pQTL from deCODE was 35,559, reflecting the number of individuals with available proteomics data from the deCODE cohort[2]. Consequently, the maximum percentage of potential sample overlap was calculated to be 2.64% for CAD (35,559 out of 1,347,212 individuals in the CAD GWAS), 2.74% for ischemic stroke (35,559 out of 1,296,908), 2.85% for cardioembolic stroke (35,559 out of 1,245,612), and 2.65% for type 2 diabetes (35,559 out of 933,970). Using the minimal F-statistics and the maximal possible sample overlap, the estimated maximum relative bias was estimated to be 0.096% for CAD, 0.100% for ischemic stroke, 0.103% for cardioembolic stroke, and 0.096% for type 2 diabetes (**Supplementary Table 10**).

Overall, due to the robust genetic instruments and minimal sample overlap, the estimated relative bias was found to be low.

Supplementary Note 2: Simulation analysis to evaluate the false positive control of two-step approach.

We conducted simulations to examine the false positive control of our two-step analytical framework. We assessed whether the association between the exposure E, e.g. BMI, and the outcome Y, e.g. coronary artery disease, could induce a spurious association between the candidate mediator M, i.e. circulating protein level, and the outcome in the absence of a true mediation effect.

We first considered two scenarios:

(1) Scenario 1: The exposure has a causal effect on the candidate mediator, while the association between the exposure and the outcome is fully due to the existence of a confounding factor U;
(2) Scenario 2: The exposure has a causal effect on the candidate mediator and an independent causal effect on the outcome.

In practice, a bi-directional relationship between the exposure and the candidate mediator is also possible. Therefore, we considered two additional scenarios:

(3) Scenario 3: The candidate mediator has a causal effect on the exposure, while the association between the exposure and the outcome is fully due to the existence of a confounding factor;
(4) Scenario 4: The candidate mediator has a causal effect on the exposure, and the exposure has a causal effect on the outcome.

We introduced a genetic instrument G for the candidate mediator, with a minor allele frequency of 0.1. Based on instrumental variable assumptions, this instrument is independent of the confounding factor and does not have any direct effects on the exposure or the outcome. Detailed model specifications are indicated in **Supplementary Fig. 1.**

We simulated each scenario 1,000 times with a sample size of 100,000. We performed MR to test whether the candidate mediator has a causal effect on the outcome in each replicate of these four scenarios.
As a result, we found that the distributions of p-values over 1,000 replicates in scenarios 1, 2 and 3 were close to a standard uniform distribution, which suggests that an effective control of false positives can be achieved with appropriate multiple testing correction. In contrast, the distribution of p-values in scenario 4 was severely left skewed. This is not surprising since the candidate mediator does have an indirect causal effect on the outcome. However, this association does not establish the mediating role of the candidate mediator, because it is the exposure that is mediating the causal effect of the candidate mediator on the outcome.
In summary, through these simulations, we have demonstrated that our two-step analytical framework is able to control for false positives, provided that valid instruments for the candidate mediator are used and that there does not exist bi-directional relationship between the exposure and the candidate mediator.

Supplementary Fig. 1. Schematic illustration of the mediation analysis and the simulation analysis.

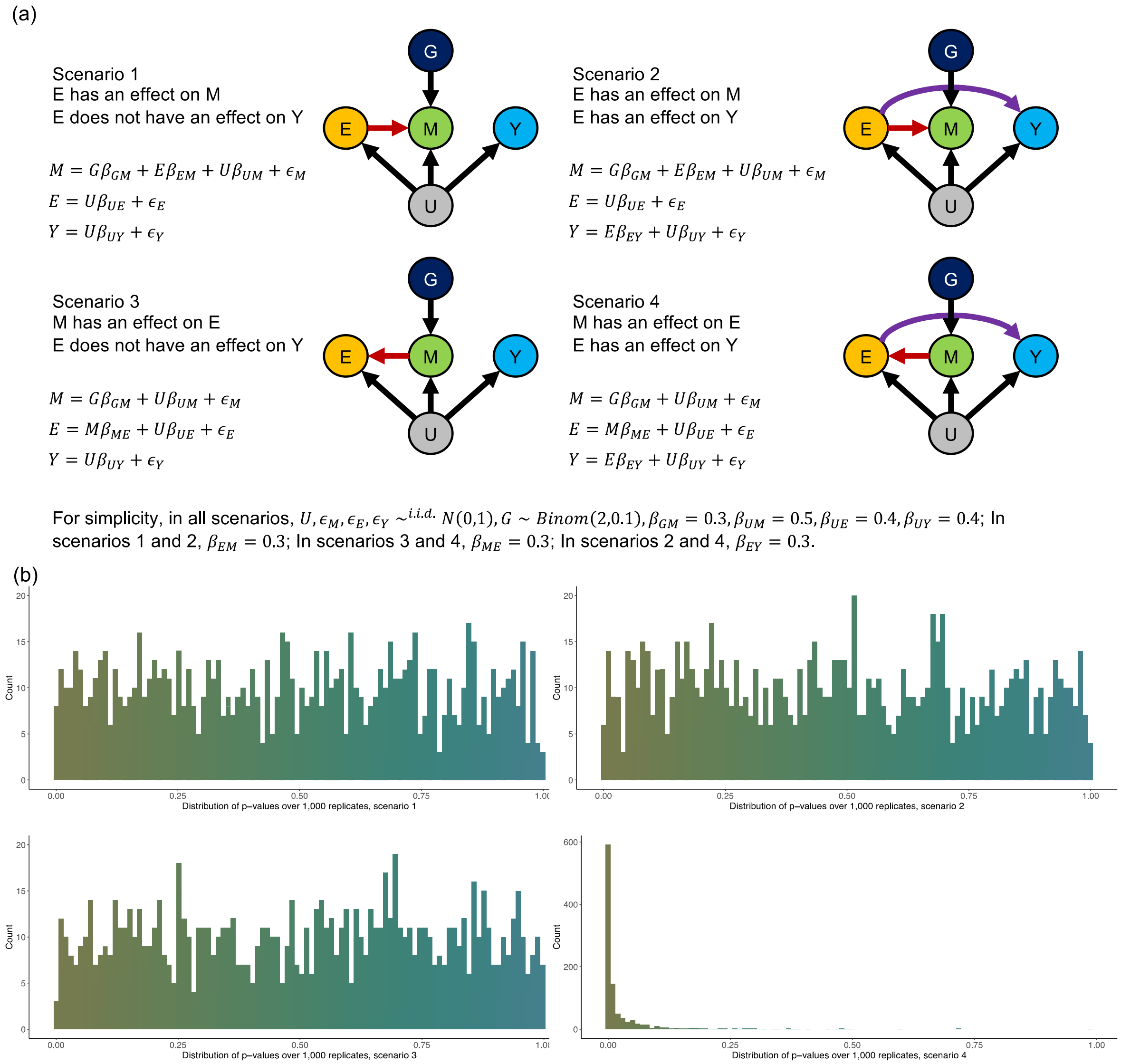

Supplementary Fig. 1. Schematic illustration of the mediation analysis and the simulation analysis.

(a) The figure demonstrates the causal relationship between BMI, the protein mediator, and cardiometabolic diseases using directed acyclic graphs. The dark blue arrow represents the total effect of BMI on cardiometabolic diseases ($\beta_{\text{BMI-to-cardiometabolic}}$), while the red arrow represents the effect of BMI on cardiometabolic diseases mediated by the protein mediator. To calculate the ratio mediated, we used the product of coefficients method. This involved multiplying the effect of BMI on the protein mediator ($\beta_{\text{BMI-to-protein}}$) by the effect of the protein mediator on cardiometabolic diseases ($\beta_{\text{protein-to-cardiometabolic}}$) to estimate the effect mediated by the protein ($\beta_{\text{mediated}} = \beta_{\text{BMI-to-protein}} \times \beta_{\text{protein-to-cardiometabolic}}$). Subsequently, we divided β_{mediated} by β_{total} to estimate the proportion mediated and calculated the *P*-value under the null hypothesis that the protein of interest did not mediate the effect of BMI on the outcome of interest (two-sided *Z* test).

BMI: body mass index, MR: Mendelian randomization.

(b) Four scenarios considered.

(c) Distributions of *P*-values (two-sided *Z* test) over 1,000 replicates in scenarios 1, 2, 3, and 4.

Supplementary Note 3: The effect of rs11677932 on Subcutaneous Adipose-Derived Mesenchymal Cells (AMSC).

Methods

We investigated the effects of the variant rs11677932 on gene expression and cellular programs in primary donor-derived differentiated subcutaneous adipose-derived mesenchymal cells (AMSC) (CellGenBank cohort)[3]. We applied both cardiometabolic disease oriented high content image-based profiling using LipocyteProfiler[3] and transcriptomics profiling which allow linking a variant of interest to gene expression changes and to annotate morphological and cellular functional effects associated with the variant. In this study, we queried donor-derived subcutaneous AMSC differentiated adipocytes and compared samples with rs11677932 homozygous reference and alternate alleles (number of samples GG=44, AA=4). We investigated the differential expression of *COL6A3* and LipocyteProfiler morphological and cellular features, where we used the non-parametric Wilcoxon comparison of means to calculate the significance of the differences associated with the reference versus alternate genotypes. The Pearson correlation was used to evaluate the correlation between the LipocyteProfiler feature and *COL6A3* expressions. In these processes, *COL6A3* expression was normalized and adjusted for patients' age, sex, BMI, and sampling batch as described previously[3]. $P < 0.05$ was considered to be nominally significant.

Results

Although the number of samples with AA genotype was low, we observed an increase, albeit non-significant, in the expression of *COL6A3* in homozygous rs11677932-G (**Supplementary Fig. 2a**). This was consistent with the direction of effect observed with *COL6A3* eQTL in aorta from GTEx v8[4], which showed rs11677932-G increases *COL6A3* expression in aorta ($\beta = 0.18$, $P = 1.6 \times 10^{-6}$).

Additionally, we investigated the effect of rs11677932-G on changes in cardiometabolic disease oriented high content imaging based cellular traits using LipocyteProfiler and found that the G allele was associated with a positive effect on the imaging feature "Nuclei AreaShape Zernike 8 2", which measures the changes in area and shape of subcutaneous adipocytes nuclei ($P = 6.1 \times 10^{-3}$) (**Supplementary Fig. 2b**). Moreover, we observed a positive trend in the relationship between the imaging feature and increased *COL6A3* expression ($r = 0.2$; $P = 0.05$) (**Supplementary Fig. 2c**).

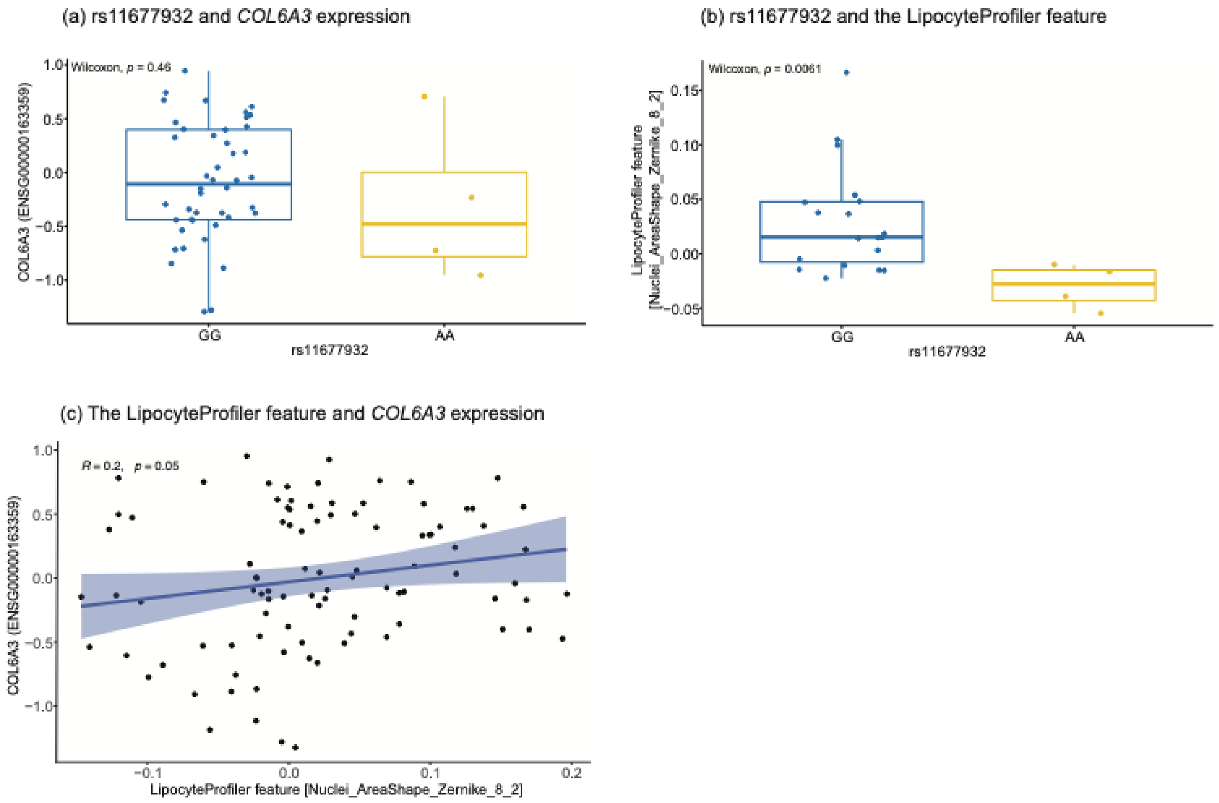

Supplementary Fig. 2. rs11677932 and LipocyteProfiler.

(a) The effect of rs11677932 on *COL6A3* expression in the subcutaneous adipose tissue. The *P*-value was obtained using a two-sided Wilcoxon rank-sum test. The boxes show the interquartile range (IQR) and horizontal lines indicating the median expression level. Whiskers represent the maximum and minimum values within 1.5 times the IQR from the first and third quartiles, and outliers are displayed as individual points.

(b) The effect of rs11677932 on the LipocyteProfiler feature "Nuclei AreaShape Zernike 8 2" in the subcutaneous adipose tissue. The *P*-value was obtained using a two-sided Wilcoxon rank-sum test. The boxes show the interquartile range (IQR) and horizontal lines indicating the median expression level. Whiskers represent the maximum and minimum values within 1.5 times the IQR from the first and third quartiles, and outliers are displayed as individual points.

(c) Association between the LipocyteProfiler feature "Nuclei AreaShape Zernike 8 2" and *COL6A3* expression. The center line represents the regression line with the shaded area indicating its 95% CI. *R* denotes Pearson's correlation coefficient. The *P*-value was obtained with two-sided Pearson's correlation test.

Supplementary Note 4: Discussion on PCSK9 and F11.

Our study replicated and highlighted other proteins, such as PCSK9 and F11. It is well established that increased plasma levels of PCSK9 elevate the risk of CAD, and inhibition of PCSK9 has been demonstrated to reduce CAD risk in multiple large clinical trials[5-7]. Additionally, a previous MR study from the INTERVAL cohort reported that BMI increases plasma PCSK9 levels, and an observational analysis in the UK Biobank study also showed that BMI is positively associated with plasma PCSK9 levels ($P < 2.2 \times 10^{-16}$)[8]. Furthermore, a 7-day caloric restriction was shown to result in decreased PCSK9 levels[9]. These reports may align with our finding that PCSK9 at least partially mediates the effect of BMI on CAD risk. However, to date, no clinical trial has specifically tested whether individuals with obesity derive greater benefit from PCSK9 inhibitors. A secondary analysis of the FOURIER randomized clinical trial[10] found that individuals with metabolic syndrome had greater absolute risk reduction (ARR) in cardiovascular events, though not reaching statistical significance. Specifically, among individuals without diabetes, those with metabolic syndrome had an ARR of 2.5% with evolocumab therapy, compared to an ARR of 0.9% in those without metabolic syndrome. Given that the trial was not powered to detect differences between these subgroups, further research is necessary to assess whether people with metabolic syndrome or obesity might benefit more from PCSK9 inhibitors in reducing CAD risk.

Another notable finding is F11 (coagulation factor XI) as a mediator of the effect of obesity on cardiometabolic disease. F11 is a critical player in the coagulation pathway and has been identified as causal for stroke by multiple studies[11,12]. However, few studies highlighted its role as a mediator. Currently, the F11 inhibitor, abelacimab[13], is in phase III clinical trial for venous thromboembolism (NCT05171049 at https://www.clinicaltrials.gov/). Our findings suggest that this drug may be effective for reducing the risk of ischemic stroke, especially for individuals with obesity.

Supplementary Note 5: Challenges regarding the estimation of the proportion mediated in MR mediation analysis with cis-pQTL.

In Step 1 MR and Step 2 MR, we showed that C-terminal COL6A3 is strongly increased by BMI and increases the risk of CAD (**Supplementary Figure 3**). However, quantifying the proportion mediated is challenging.

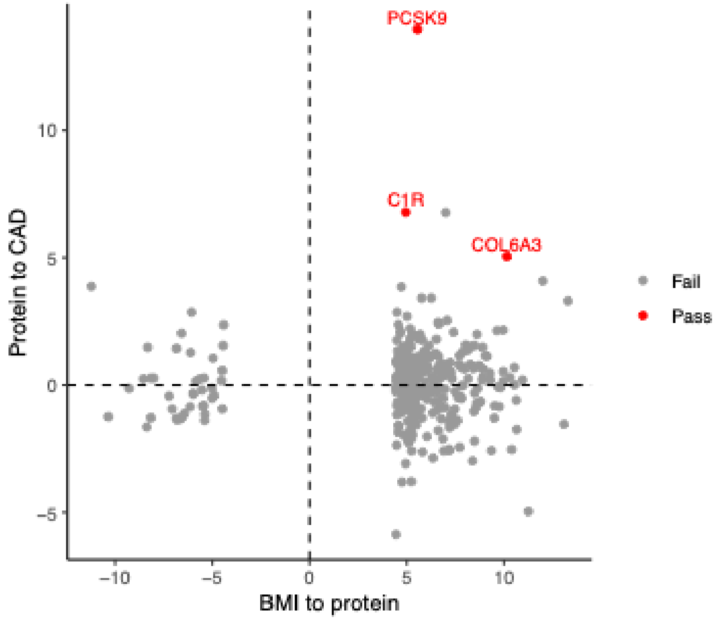

Supplementary Figure 3. The scatter plot of z-scores from Step 1 MR and Step 2 MR. The x-axis represents each protein's z-score from Step 1 MR, where we estimated the effect of BMI on the plasma level of each protein. The y-axis represents each protein's z-score from Step 2 MR (Protein-to-CAD), where we estimated the effect of the corresponding proteins on CAD risk. Red circles represent proteins that passed both Step 1 and Step 2 MR, while grey circles represent proteins that passed Step 1 MR but failed Step 2 MR.

Traditional non-instrumental variable (IV) mediation analysis relies on strong assumptions. Some of which are untestable, such as (i) no unmeasured confounding between the exposure, mediator, and outcome; (ii) no exposure-caused confounders of the mediator-outcome relationship; and (iii) no exposure-mediator interaction. MR can be used to overcome some of these challenges[14-16]. Importantly, MR estimates are less likely to be biased due to unmeasured confounding among the exposure, mediator, or outcome. However, it still relies on multiple assumptions: it assumes no interaction between the exposure and mediator and that instrumental variables influence the outcome solely through the exposure and mediator (exclusion restriction), in addition to all the other MR assumptions.

Currently, there are two methods to estimate the proportion mediated in MR mediation analysis: (i) the multivariable MR (MVMR) approach and (ii) the product of coefficients method (network MR approach)[14]. In the MVMR approach, the direct effect of the exposure on the outcome, controlling for the mediator, is estimated by including genetic instruments for both the primary exposure and the mediator. For the product of coefficients method (network MR approach), MVMR is still required when estimating the causal effect of the mediator (C-terminal COL6A3) on the outcome (CAD), adjusting for the primary exposure (BMI) (**Supplementary Fig. 4a**).

However, using *cis*-pQTL of C-terminal COL6A3 in MVMR, with protein level and BMI as exposures, poses unique challenges due to the distinct genetic architectures: the highly polygenic nature of BMI with widespread associations across the genome and much simpler genetic architecture of pQTL with strong associations in the *cis*-region for the protein level.

There are only up to two instrumental variables for C-terminal COL6A3 depending on the cohorts (two from the deCODE cohort; one from other cohorts), while there are 304 instrumental variables for BMI across the genome, reflecting its polygenic nature. Specifically, there is no instrumental variable for BMI in the cis-region of the COL6A3 gene. This means that including instrumental variables for BMI and cis-pQTL in the same MVMR is likely to introduce weak instrumental bias and horizontal pleiotropy. For example, adding the instrumental variable of BMI to cis-pQTL of C-terminal COL6A3 will "dilute" the strong association between the cis-pQTL and its protein levels (see **Supplementary Fig. 4b**).

Currently, there is no effective method to overcome the above-mentioned challenges. Therefore, further method development or refinement is required to conduct multivariable MR using cis-pQTL (or any cis molecular trait) and instrumental variables of polygenic traits such as BMI.

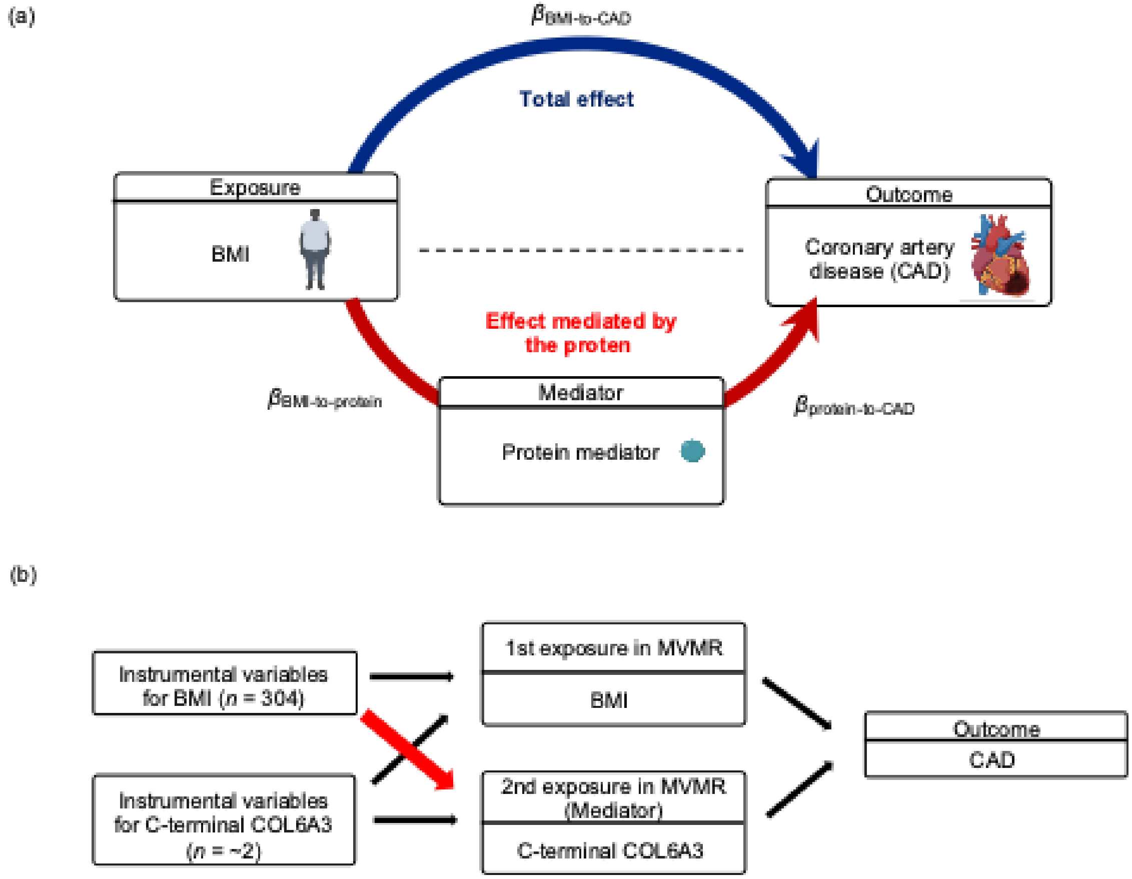

Supplementary Fig. 4. Schematic illustration of a product of coefficients method for MR mediation analysis and multivariable MR.
(a) The figure demonstrates the causal relationship between BMI, the protein mediator, and cardiometabolic diseases using directed acyclic graphs. The dark blue arrow represents the total effect of BMI on cardiometabolic diseases ($\beta_{\text{BMI-to-CAD}}$), while the red arrow represents the effect of

BMI on cardiometabolic diseases mediated by the protein mediator ($\beta_{mediated} = \beta_{BMI\text{-}to\text{-}protein} \times \beta_{protein\text{-}to\text{-}CAD}$).
(b) The genetic instrumental variables (IVs) for BMI will "dilute" the association between the IV and C-terminal COL6A3 level in MVMR. The red arrow represents the association between IV for BMI and C-terminal COL6A3 level, which may introduce the weak instrumental bias and horizontal pleiotropy.

Network MR without MVMR (without adjustment for BMI)

One way to bypass the issue mentioned above relating to MVMR is to use the product-of-coefficients method without adjusting for BMI when estimating the effect of C-terminal COL6A3 (the mediator) on CAD risk (the outcome) ($\beta_{protein\text{-}to\text{-}CAD}$ in **Supplementary Figure 3**). This method has been used in multiple studies[14,17,18]. However, it is important to note that this approach still relies on all the aforementioned assumptions and may inflate the estimated proportion mediated due to a larger $\beta_{protein\text{-}to\text{-}CAD}$. Nevertheless, as a supplementary analysis, we applied this method to estimate the proportion of the total effect of BMI on CAD risk mediated by the C-terminal COL6A3 level.

Methods:

To estimate the causal mediation effects ($\beta_{mediated}$), we estimated the effect of BMI on the plasma protein levels ($\beta_{BMI\text{-}to\text{-}protein}$) and the effect of the plasma proteins on cardiometabolic diseases ($\beta_{protein\text{-}to\text{-}cardiometabolic}$), and then multiplied these values ($\beta_{mediated} = \beta_{BMI\text{-}to\text{-}protein} \times \beta_{protein\text{-}to\text{-}CAD}$). For this, we performed MR using the same instrumental variables as in Steps 1 and 2 of MR. Subsequently, we divided $\beta_{mediated}$ by β_{total} to estimate the proportion mediated and calculated the *P*-value under the null hypothesis that the protein of interest did not mediate the effect of BMI on the outcome of interest. We considered a result with $P < 0.05$ to be significant (denoted with an asterisk *).

Results:

Below are the estimated effects of BMI on CAD ($\beta_{BMI\text{-}to\text{-}CAD}$), BMI to C-COL6A3 ($\beta_{BMI\text{-}to\text{-}protein}$), and C-COL6A3 to CAD ($\beta_{protein\text{-}to\text{-}CAD}$). The estimated proportion mediated was 32.2% (95% CI: 17.2%–47.1%), $P = 2.54 \times 10^{-5}$.

MR (exposure-to-outcome)	method	SNPs	Estimate	95% CI Lower	95% CI Upper	*P*
BMI to the CAD ($\beta_{BMI\text{-}to\text{-}CAD}$)	IVW	294	0.38 (0.32–0.45)	0.32	0.45	4.16×10^{-31}
BMI to C-COL6A3 ($\beta_{BMI\text{-}to\text{-}protein}$)	IVW	285	0.32 (0.26–0.38)	0.26	0.38	3.65×10^{-24}
C-COL6A3 to CAD ($\beta_{protein\text{-}to\text{-}CAD}$)	Wald ratio	1	0.38 (0.23–0.53)	0.23	0.53	4.46×10^{-7}
Proportion mediated	–	–	32.2%	17.2%	47.1%	2.54×10^{-5}*.

Observational mediation analysis in the EPIC-Norfolk cohort

Although observational mediation analysis relies on strong assumptions, as additional supplementary analysis, we performed observational mediation analysis using individual-level data from the EPIC-Norfolk cohort (see ***Observational regression analysis with individual-level data in the EPIC-Norfolk cohort*** in **Methods** for further details of the cohort).

Methods:

We used the product of coefficients methods to calculate the proportion mediated, as described above, using the R package mediation v4.5.0. We used linear regression analysis to estimate the effect of BMI on plasma C-terminal COL6A3 levels. We used logistic regression to estimate the effect of BMI on the CAD risk and the effect of C-terminal COL6A3 levels on the CAD risk. For covariates, we adjusted for age and sex given the availability of data. Significance of the indirect effect and the proportion mediated was estimated by computing unstandardized effects in 1,000 bootstrapped samples and calculating the corresponding 95% confidence intervals.

Results: Proportion mediated was estimated to be 20.54% (95% CI: 3.48%–81%), $P = 0.018$.

Mediation analyses in the EPIC-Norfolk cohort				
Effect	Estimate	95% CI Lower	95% CI Upper	*P*
BMI-to-COL6A3 (β, linear regression)	0.060	0.043	0.08	8.54×10^{-12}
COL6A3-to-CAD (odds ratio, logistic regression)	1.34	1.12	1.59	1.12×10^{-3}
BMI-to-CAD (odds ratio, logistic regression)	0.0035	0.0018	0	0.006
Proportion mediated	20.54%	3.48%	81%	0.018*

Observational mediation analysis in the UK Biobank

We repeated the observational mediation analysis in 35,100 individuals from the UK Biobank. We included participants from the UK Biobank for whom we have protein measurements with the Olink Explore 3072 assay (UK Biobank data field: 30900) and ICD10-based diagnosis (data field: 41270). Further details of the cohort can be found in the **Methods** (see ***Observational regression analysis for baseline BMI and plasma C-terminal COL6A3 level in the UK Biobank***). We adjusted for age and sex and used the R package mediation v4.5.0, as described above.

Estimated proportion mediated was 35.34% (95% CI: 28.6%–44%, $P < 2.2 \times 10^{-16}$)

Effect	Estimate	95% CI Lower	95% CI Upper	*P*
BMI-to-COL6A3 (β, linear regression)	0.060	0.043	0.08	8.54×10^{-12}
COL6A3-to-CAD (odds ratio, logistic regression)	1.34	1.12	1.59	1.12×10^{-3}
BMI-to-CAD (odds ratio, logistic regression)	0.0035	0.0018	0	0.006
Proportion mediated	35.34%	28.6%	44%	$<2.2 \times 10^{-16*}$

We note that these estimates of three methods/datasets align with each other: The estimated proportion mediated from the MR mediation analysis = 32.2% (95% CI: 17.2%–47.1%), $P = 2.54 \times 10^{-5}$; that from the observational analysis in the EPIC-Norfolk = 20.54% (95% CI: 3.48%–81%), $P = 0.018$; that from the observational analysis in the UK Biobank = 35.34% (95%CI: 28.6%–44%), $P < 2.2 \times 10^{-16}$.

The high estimated proportion mediated may reflect the fact that (i) C-terminal COL6A3/endotrophin plays an important role in adipocyte fibrosis and insulin resistance[19-22]; and (ii) we did not model all causal proteins that play a role in these pathways, which means that some of the causal effect attributed to C-terminal COL6A3 may be shared with other proteins. Jointly modeling all causal protein mediators could reveal the proportion mediated exclusive to each protein but requires using MVMR with *cis*-MR, which is challenging for the reasons mentioned above. Importantly, it was not possible to adjust for BMI when estimating the effect of C-terminal

COL6A3 on CAD ($\beta_{\text{protein-to-CAD}}$), which again requires MVMR with *cis*-MR. To overcome these limitations, further method development or refinement is required.

Supplementary Note 6: STROBE-MR checklist of recommended items to address in reports of Mendelian randomization studies.

Item No.	Section	Checklist item	Relevant text from manuscript
1	TITLE and ABSTRACT	Indicate Mendelian randomization (MR) as the study's design in the title and/or the abstract if that is a main purpose of the study	Specified in the abstract.
	INTRODUCTION		
2	Background	Explain the scientific background and rationale for the reported study. What is the exposure? Is a potential causal relationship between exposure and outcome plausible? Justify why MR is a helpful method to address the study question	Explained in the Introduction section.
3	Objectives	State specific objectives clearly, including pre-specified causal hypotheses (if any). State that MR is a method that, under specific assumptions, intends to estimate causal effects	Explained in the last paragraph of the Background section.
	METHODS		
4	Study design and data sources	Present key elements of the study design early in the article. Consider including a table listing sources of data for all phases of the study. For each data source contributing to the analysis, describe the following:	Explained in the Results and Methods sections and sources of data is presented in supplementary table 1 (ST1).
	a)	Setting: Describe the study design and the underlying population, if possible. Describe the setting, locations, and relevant dates, including periods of recruitment, exposure, follow-up, and data collection, when available.	The study design and the underlying population is described in ST1 and Methods section.
	b)	Participants: Give the eligibility criteria, and the sources and methods of selection of participants. Report the sample size, and whether any power or sample size calculations were carried out prior to the main analysis	(b)–(e) were described in the Methods section.
	c)	Describe measurement, quality control and selection of genetic variants	
	d)	For each exposure, outcome, and other relevant variables, describe methods of assessment and diagnostic criteria for diseases	
	e)	Provide details of ethics committee approval and participant informed consent, if relevant	
5	Assumptions	Explicitly state the three core IV assumptions for the main analysis (relevance, independence and exclusion restriction) as well assumptions for any additional or sensitivity analysis	Explicitly stated in the Methods.
6	Statistical methods: main analysis	Describe statistical methods and statistics used	
	a)	Describe how quantitative variables were handled in the analyses (i.e., scale, units, model)	(a)–(e) were described in the Methods as well as the Results section.
	b)	Describe how genetic variants were handled in the analyses and, if applicable, how their weights were selected	
	c)	Describe the MR estimator (e.g. two-stage least squares, Wald ratio) and related statistics. Detail the included covariates and, in case of two-sample MR, whether the same covariate set was used for adjustment in the two samples	
	d)	Explain how missing data were addressed	
	e)	If applicable, indicate how multiple testing was addressed	
7	Assessment of assumptions	Describe any methods or prior knowledge used to assess the assumptions or justify their validity	(a)–(e) were described in the Methods as well as the Results section.
8	Sensitivity analyses and additional analyses	Describe any sensitivity analyses or additional analyses performed (e.g. comparison of effect estimates from different approaches, independent replication, bias analytic techniques, validation of instruments, simulations)	
9	Software and pre-registration		
	a)	Name statistical software and package(s), including version and settings used	
	b)	State whether the study protocol and details were pre-registered (as well as when and where)	
	RESULTS		

10	Descriptive data		
	a)	Report the numbers of individuals at each stage of included studies and reasons for exclusion. Consider use of a flow diagram	Described in the Methods and Supplementary Table 1.
	b)	Report summary statistics for phenotypic exposure(s), outcome(s), and other relevant variables (e.g. means, SDs, proportions)	Described in the Methods and Supplementary Table 1.
	c)	If the data sources include meta-analyses of previous studies, provide the assessments of heterogeneity across these studies	Discussed in the original papers. We also evaluated the heterogeneity and horizontal pleiotropy in our analyses.
	d)	For two-sample MR:<br>i. Provide justification of the similarity of the genetic variant-exposure associations between the exposure and outcome samples<br>ii. Provide information on the number of individuals who overlap between the exposure and outcome studies	Described in the Methods, Results, and Supplementary Note.
11	Main results		
	a)	Report the associations between genetic variant and exposure, and between genetic variant and outcome, preferably on an interpretable scale	a)–(c) were described in the Results.
	b)	Report MR estimates of the relationship between exposure and outcome, and the measures of uncertainty from the MR analysis, on an interpretable scale, such as odds ratio or relative risk per SD difference	
	c)	If relevant, consider translating estimates of relative risk into absolute risk for a meaningful time period	
	d)	Consider plots to visualize results (e.g. forest plot, scatterplot of associations between genetic variants and outcome versus between genetic variants and exposure)	Described in the Methods, Results, and Figures with a scatter plot.
12	Assessment of assumptions		
	a)	Report the assessment of the validity of the assumptions	Described in the Methods and Results.
	b)	Report any additional statistics (e.g., assessments of heterogeneity across genetic variants, such as I^2, Q statistic or E-value)	Described in the Methods and Results.
13	Sensitivity analyses and additional analyses		
	a)	Report any sensitivity analyses to assess the robustness of the main results to violations of the assumptions	(a)-(d) were described in the Methods and Results.
	b)	Report results from other sensitivity analyses or additional analyses	
	c)	Report any assessment of direction of causal relationship (e.g., bidirectional MR)	
	d)	When relevant, report and compare with estimates from non-MR analyses	
	e)	Consider additional plots to visualize results (e.g., leave-one-out analyses)	We did not perform leave-one-out analyses; instead, we assessed the robustness of the analyses using an alternative outlier-removal method (MR-PRESSO), which is described in the Methods and Results sections.
	DISCUSSION		
14	Key results	Summarize key results with reference to study objectives	Described in the Discussion section.
15	Limitations	Discuss limitations of the study, taking into account the validity of the IV assumptions, other sources of potential bias, and imprecision.	Described in the Discussion section.

		Discuss both direction and magnitude of any potential bias and any efforts to address them	
16	Interpretation		
	a)	Meaning: Give a cautious overall interpretation of results in the context of their limitations and in comparison with other studies	(a)–(c) were described in the Results and Discussion sections.
	b)	Mechanism: Discuss underlying biological mechanisms that could drive a potential causal relationship between the investigated exposure and the outcome, and whether the gene-environment equivalence assumption is reasonable. Use causal language carefully, clarifying that IV estimates may provide causal effects only under certain assumptions	
	c)	Clinical relevance: Discuss whether the results have clinical or public policy relevance, and to what extent they inform effect sizes of possible interventions	
17	Generalizability	Discuss the generalizability of the study results (a) to other populations, (b) across other exposure periods/timings, and (c) across other levels of exposure	Described in the Discussion section.
	OTHER INFORMATION		
18	Funding	Describe sources of funding and the role of funders in the present study and, if applicable, sources of funding for the databases and original study or studies on which the present study is based	Described in the Acknowledgments.
19	Data and data sharing	Provide the data used to perform all analyses or report where and how the data can be accessed, and reference these sources in the article. Provide the statistical code needed to reproduce the results in the article, or report whether the code is publicly accessible and if so, where	Described in the Data Availability and Code availability.
20	Conflicts of Interest	All authors should declare all potential conflicts of interest	Described in the Competing Interests.

Supplementary Note 7: Definition of coronary artery disease in the EPIC-Norfolk analysis.

The EPIC-Norfolk study, a component of the pan-European EPIC Study, is a cohort of 25,639 middle-aged individuals from the general population of Norfolk, a county in Eastern England[23], who attended the baseline assessment between 1993–1998. We performed observational regression analysis in a randomly selected subcohort (*n* = 872) of the EPIC-Norfolk study, in which proteomic profiling was performed using the SomaScan v.4 assay. Death certificates and hospitalization data were obtained using National Health Service (NHS) numbers through linkage with the NHS digital database. Electronic health records were coded by trained nosologists according to the International Statistical Classification of Diseases and Related Health Problems, 9th (ICD-9) or 10th Revision (ICD-10). Participants were identified as CAD cases if the corresponding ICD-codes (ICD-9: 410-414, ICD-10: I20-I25) were registered on the death certificate (as the underlying cause of death or as a contributing factor), or as the cause of hospitalization. The current study is based on follow-up to the 31st March 2018. The case definition included all individuals identified as prevalent (at the baseline study assessment) or incident CAD cases over the follow-up period of over 20 years.

Supplementary Note 8: GWAS of the sex-stratified pQTL for C-terminal COL6A3.

Proteomics data were pre-processed and underwent quality control by the UKB-PPP. GWAS was performed using REGENIE v3.2.9, which consists of two steps[24]. In the first step, we used high quality variants that underwent stringent filtering to fit the whole genome model. The filtering criteria included MAF > 1%, MAC > 100, genotype rate > 99%, Hardy–Weinberg equilibrium test $P > 10^{-15}$, < 10% missingness, and LD pruning (1,000 variant windows, 100 sliding windows and $r^2 < 0.8$. We excluded individuals whose genetic sex did not match their reported sex, as well as those whose proteins were measured in Olink batch 0 or Olink batch 7 (COVID-19 imaging study), focusing on Olink batches 1–6. In the second step, genetic association analyses were performed with plasma levels of C-terminal COL6A3 conditional upon the prediction from the regression model in step 1. We limited genetic association analyses to variants with INFO > 0.7 and MAC > 50 to minimize spurious associations. We restricted the analysis to those whose genetic grouping is European ancestry (UK Biobank Data-Field 22006). For covariates, we included age at recruitment (age), age*age, time between sample collection, genotype measurement batch, 20 principal components, as well as the Olink batch number and recruitment center, as done previously[8].

Supplementary Note 9: Quality control in the single-cell RNA sequencing analysis.

Following Wirka et al.[25], we removed low-quality cells that expressed < 500 genes or had a mitochondrial content > 7.5%, and genes expressed in < 5 cells. Cells expressing > 3,500 genes were also removed to avoid bias due to doublets. The retained gene expression profiles were normalized to library size. The top 2,000 most variable genes were selected after variance-stabilizing transformation using the FindVariableFeatures function in Seurat v4.0.6. Principal component analysis was performed based on these 2,000 most variable genes after scaling and centering. Nearest-neighbor graph construction was conducted based on the first 10 principal components using the FindNeighbors function in Seurat v4.0.6 with default settings. Cell clusters were identified using the FindClusters function in Seurat v4.0.6 with default settings. Uniform Manifold Approximation and Projection (UMAP) was also performed on the first 10 principal components. Two-dimensional visualization of the cell clusters was based on the first two UMAP dimensions.

References:

1 Burgess, S., Davies, N. M. & Thompson, S. G. Bias due to participant overlap in two-sample Mendelian randomization. *Genet. Epidemiol.* **40**, 597-608 (2016). https://doi.org/10.1002/gepi.21998

2 Ferkingstad, E. *et al.* Large-scale integration of the plasma proteome with genetics and disease. *Nat. Genet.* **53**, 1712-1721 (2021). https://doi.org/10.1038/s41588-021-00978-w

3 Laber, S. *et al.* Discovering cellular programs of intrinsic and extrinsic drivers of metabolic traits using LipocyteProfiler. *Cell Genom* **3**, 100346 (2023). https://doi.org/10.1016/j.xgen.2023.100346

4 GTExConsortium. The GTEx Consortium atlas of genetic regulatoryeffects across human tissues. *Science* **369**, 1318-1330 (2020).

5 Sabatine, M. S. *et al.* Evolocumab and Clinical Outcomes in Patients with Cardiovascular Disease. *N. Engl. J. Med.* **376**, 1713-1722 (2017). https://doi.org/10.1056/NEJMoa1615664

6 Schwartz, G. G. *et al.* Alirocumab and Cardiovascular Outcomes after Acute Coronary Syndrome. *N. Engl. J. Med.* **379**, 2097-2107 (2018). https://doi.org/10.1056/NEJMoa1801174

7 Ray, K. K. *et al.* Two Phase 3 Trials of Inclisiran in Patients with Elevated LDL Cholesterol. *N. Engl. J. Med.* **382**, 1507-1519 (2020). https://doi.org/10.1056/NEJMoa1912387

8 Sun, B. B. *et al.* Plasma proteomic associations with genetics and health in the UK Biobank. *Nature* **622**, 329-338 (2023). https://doi.org/10.1038/s41586-023-06592-6

9 Pietzner, M. *et al.* Systemic proteome adaptions to 7-day complete caloric restriction in humans. *Nature Metabolism* (2024). https://doi.org/10.1038/s42255-024-01008-9

10 Deedwania, P. *et al.* Efficacy and Safety of PCSK9 Inhibition With Evolocumab in Reducing Cardiovascular Events in Patients With Metabolic Syndrome Receiving Statin Therapy: Secondary Analysis From the FOURIER Randomized Clinical Trial. *JAMA Cardiol* **6**, 139-147 (2021). https://doi.org/10.1001/jamacardio.2020.3151

11 Zheng, J. *et al.* Phenome-wide Mendelian randomization mapping the influence of the plasma proteome on complex diseases. *Nat. Genet.* **52**, 1122-1131 (2020). https://doi.org/10.1038/s41588-020-0682-6

12 Georgakis, M. K. & Gill, D. Mendelian Randomization Studies in Stroke: Exploration of Risk Factors and Drug Targets With Human Genetic Data. *Stroke* **52**, 2992-3003 (2021). https://doi.org/10.1161/STROKEAHA.120.032617

13 Verhamme, P. *et al.* Abelacimab for Prevention of Venous Thromboembolism. *N. Engl. J. Med.* **385**, 609-617 (2021). https://doi.org/10.1056/NEJMoa2105872

14 Carter, A. R. *et al.* Mendelian randomisation for mediation analysis: current methods and challenges for implementation. *Eur. J. Epidemiol.* **36**, 465-478 (2021). https://doi.org/10.1007/s10654-021-00757-1

15 Sanderson, E. Multivariable Mendelian Randomization and Mediation. *Cold Spring Harb. Perspect. Med.* **11** (2021). https://doi.org/10.1101/cshperspect.a038984

16 Richmond, R. C., Hemani, G., Tilling, K., Davey Smith, G. & Relton, C. L. Challenges and novel approaches for investigating molecular mediation. *Hum. Mol. Genet.* **25**, R149-R156 (2016). https://doi.org/10.1093/hmg/ddw197

17 Burgess, S., Daniel, R. M., Butterworth, A. S., Thompson, S. G. & Consortium, E. P.-I. Network Mendelian randomization: using genetic variants as instrumental variables to investigate mediation in causal pathways. *Int. J. Epidemiol.* **44**, 484-495 (2015). https://doi.org/10.1093/ije/dyu176

18 Woolf, B., Zagkos, L. & Gill, D. TwoStepCisMR: A Novel Method and R Package for Attenuating Bias in cis-Mendelian Randomization Analyses. *Genes* **13** (2022). https://doi.org/10.3390/genes13091541
19 Sun, K. *et al.* Endotrophin triggers adipose tissue fibrosis and metabolic dysfunction. *Nat. Commun.* **5**, 3485 (2014). https://doi.org/10.1038/ncomms4485
20 Jo, W. *et al.* MicroRNA-29 Ameliorates Fibro-Inflammation and Insulin Resistance in HIF1alpha-Deficient Obese Adipose Tissue by Inhibiting Endotrophin Generation. *Diabetes* **71**, 1746-1762 (2022). https://doi.org/10.2337/db21-0801
21 Williams, L., Layton, T., Yang, N., Feldmann, M. & Nanchahal, J. Collagen VI as a driver and disease biomarker in human fibrosis. *FEBS J.* **289**, 3603-3629 (2022). https://doi.org/10.1111/febs.16039
22 Li, X. *et al.* Critical Role of Matrix Metalloproteinase 14 in Adipose Tissue Remodeling during Obesity. *Mol. Cell. Biol.* **40**, e00564-00519 (2020). https://doi.org/doi:10.1128/MCB.00564-19
23 Day, N. *et al.* EPIC-Norfolk: study design and characteristics of the cohort. European Prospective Investigation of Cancer. *Br. J. Cancer* **80 Suppl 1**, 95-103 (1999).
24 Mbatchou, J. *et al.* Computationally efficient whole-genome regression for quantitative and binary traits. *Nat. Genet.* (2021). https://doi.org/10.1038/s41588-021-00870-7
25 Wirka, R. C. *et al.* Atheroprotective roles of smooth muscle cell phenotypic modulation and the TCF21 disease gene as revealed by single-cell analysis. *Nat. Med.* **25**, 1280-1289 (2019). https://doi.org/10.1038/s41591-019-0512-5
