# Supplementary material for: Integrative proteogenomic analysis identifies COL6A3-derived endotrophin as a mediator of the effect of obesity on coronary artery disease
Source: Nat Genet. 2025 Jan 24;57(2):345–57. doi: 10.1038/s41588-024-02052-7 (PMC11821532; doi:10.1038/s41588-024-02052-7)
Supplement: Supplementary file 2 — Reporting Summary [file 41588_2024_2052_MOESM2_ESM.pdf]

nature portfolio

Corresponding author(s): J. Brent Richards

Last updated by author(s): Nov 5, 2024

Reporting Summary

Nature Portfolio wishes to improve the reproducibility of the work that we publish. This form provides structure for consistency and transparency in reporting. For further information on Nature Portfolio policies, see our Editorial Policies and the Editorial Policy Checklist.

Statistics

For all statistical analyses, confirm that the following items are present in the figure legend, table legend, main text, or Methods section.

n/a	Confirmed	
☐	☒	The exact sample size (n) for each experimental group/condition, given as a discrete number and unit of measurement
☐	☒	A statement on whether measurements were taken from distinct samples or whether the same sample was measured repeatedly
☐	☒	The statistical test(s) used AND whether they are one- or two-sided<br>*Only common tests should be described solely by name; describe more complex techniques in the Methods section.*
☐	☒	A description of all covariates tested
☐	☒	A description of any assumptions or corrections, such as tests of normality and adjustment for multiple comparisons
☐	☒	A full description of the statistical parameters including central tendency (e.g. means) or other basic estimates (e.g. regression coefficient) AND variation (e.g. standard deviation) or associated estimates of uncertainty (e.g. confidence intervals)
☐	☒	For null hypothesis testing, the test statistic (e.g. F, t, r) with confidence intervals, effect sizes, degrees of freedom and P value noted<br>*Give P values as exact values whenever suitable.*
☒	☐	For Bayesian analysis, information on the choice of priors and Markov chain Monte Carlo settings
☒	☐	For hierarchical and complex designs, identification of the appropriate level for tests and full reporting of outcomes
☐	☒	Estimates of effect sizes (e.g. Cohen's d, Pearson's r), indicating how they were calculated

Our web collection on statistics for biologists contains articles on many of the points above.

Software and code

Policy information about availability of computer code

Data collection	No software has been used for data acquisition.
Data analysis	We used R v4.1.2 (https://www.r-project.org/), TwoSampleMR v.0.5.6 (https://mrcieu.github.io/TwoSampleMR/), survival v3.5.8 (https://github.com/therneau/survival), trackplot R v1.0 (https://github.com/PoisonAlien/trackplot), RNOmni v1.01 (https://github.com/zrmacc/RNOmni), snappy v1.0 (https://gitlab.com/richardslab/vince.forgetta/snappy), coloc v5.1.0 (https://chr1swallace.github.io/coloc/), PLINK v1.9 (http://pngu.mgh.harvard.edu/purcell/plink/), susieR v 0.11.92 (https://github.com/stephenslab/susieR/), REGENIE v3.2.9 (https://github.com/rgcgithub/regenie/), and Seurat v4.0.6 (https://satijalab.org/seurat/). Custom codes are available on GitHub (https://github.com/satoshiyoshiji/cm_proteogenomics/).

For manuscripts utilizing custom algorithms or software that are central to the research but not yet described in published literature, software must be made available to editors and reviewers. We strongly encourage code deposition in a community repository (e.g. GitHub). See the Nature Portfolio guidelines for submitting code & software for further information.

Data

Policy information about availability of data

All manuscripts must include a data availability statement. This statement should provide the following information, where applicable:

- Accession codes, unique identifiers, or web links for publicly available datasets
- A description of any restrictions on data availability
- For clinical datasets or third party data, please ensure that the statement adheres to our policy

The GWAS of plasma COL6A3 level in males and females are available at GWAS Catalog (GCP ID: GCP001023). We used publicly available GWAS summary statistics from the following source:
BMI GWAS from GIANT and UK Biobank (https://portals.broadinstitute.org/collaboration/giant/; doi: 10.1093/hmg/ddy271).
Plasma proteome GWAS from deCODE (https://www.deCODE.com/summarydata/; doi: 10.1038/s41588-021-00978-w), UK Biobank (https://www.ukbiobank.ac.uk/; doi: 10.1101/2022.06.17.496443), Fenland (https://omicscience.org/apps/pgwas/; doi: 10.1126/science.abj1541), and ARIC (http://nilanjanchatterjeelab.org/pwas/; doi: 10.1038/s41588-022-01051-w).
We also used the CAD GWAS from CARDIoGRAMplusC4D (http://www.cardiogramplusc4d.org/ doi: 10.1038/s41588-022-01233-6), stroke GWAS from GIGASTROKE (GCST90104534 and GCST90104535, at https://www.ebi.ac.uk/gwas/studies/), and type 2 diabetes GWAS from Mahajan et al. (https://doi.org/10.1038/s41588-022-01058-3).
For gene expression data, we used data from Nathan et al. (SCP498 at Single Cell Portal https://singlecell.broadinstitute.org/) and Wirka et al (GSE131780 at Gene Expression Omnibus database https://www.ncbi.nlm.nih.gov/geo/).
Epigenomic data are available at RegulomeDB (https://regulomedb.org/) and ENCODE (https://www.encodeproject.org/).
Variant-to-gene (V2G) scores are available at the Open Target Genetics (https://genetics-docs.opentargets.org/data-access/data-download).
Individual-level data of the UK Biobank, EPIC-Norfolk, and CellGenBankCohort are available through respective party upon agreement.

Research involving human participants, their data, or biological material

Policy information about studies with human participants or human data. See also policy information about sex, gender (identity/presentation), and sexual orientation and race, ethnicity and racism.

Reporting on sex and gender	We performed sex-stratified analyses based on genetically determined sex (UK Biobank data-field 22001).
Reporting on race, ethnicity, or other socially relevant groupings	We focused on analyzing data solely from European-ancestry individuals to prevent confounding by population stratification. While the ARIC cohort reported cis-pQTL for individuals of African ancestry16, the sample size (n = 1,871) is still limited when compared to data for those of European ancestry (deCODE study; n = 35,559). The same applies to CAD GWAS, with 181,522 CAD cases in European ancestry individuals compared to only 17,247 cases in African ancestry individuals. This limited sample size in African ancestry individuals reduces the statistical power of MR analysis. Therefore, further efforts are needed to increase the sample size of non-European-ancestry data.
Population characteristics	•BMI GWAS: We used the BMI GWAS meta-analysis with the largest sample size, comprising 693,529 European ancestry individuals from the GIANT consortium and UK Biobank.<br>•Body fat percentage GWAS: We used body fat percentage GWAS in 454,633 individuals of European ancestry from the UK Biobank, obtained from the IEU OpenGWAS project (https://gwas.mrcieu.ac.uk/). The accession ID was ukb-b-8909.<br>•Proteomic GWAS: For the primary analysis, we used the largest proteomic GWAS available, which measured 4,907 proteins in 35,559 individuals of European ancestry from the deCODE study (Ferkingstad et al.). We also used the GWAS of plasma COL6A3 level from UK Biobank in 35,571 individuals of European ancestry, that from the Fenland in 12,084 individuals of European ancestry, and that from ARIC in individuals of European ancestry in 7,213 individuals of Icelandic ancestry.<br><br>•Coronary artery disease GWAS: Meta-analysis of GWASs in individuals of European ancestry (181,522 cases and 984,168 controls) from UK Biobank + CARDIoGRAMplusC4D (Aragam et al. Nat Genet 2022).<br>•Ischemic stroke GWAS: Meta-analysis of GWASs in individuals of European ancestry (73,652 cases and 1,234,808 controls) from GIGASTROKE (Mishra et al. Nature 2022).<br>•Cardioembolic stroke GWAS: Meta-analysis of GWASs in individuals of European ancestry (122,616 cases and 2,475,240 controls) from GIGASTROKE (Mishra et al. Nature 2022).<br>•Type 2 diabetes GWAS: Meta-analysis of GWASs in individuals of European ancestry (80,154 cases and 853,816 controls) from DIAMANTE (Mahajan et al. Nat Genet 2022).<br>•EPIC-Norfolk: The EPIC-Norfolk study is a component of the pan-European EPIC Study, a population-based cohort in Norfolk, a county in Eastern England. We performed observational association analysis with a randomly selected sub-cohort of the EPIC-Norfolk study (n = 872), which included 207 prevalent or incident cases of CAD. Mean age = 59.05 (SD = 9.54). N females = 502 (57.6%).<br>•UK Biobank: The UK Biobank is a large-scale population-based prospective study. We performed Cox regression analysis using individual-level data from 38,361 people, which included baseline clinical variables, C-terminal COL6A3 levels, and cumulative CAD events over up to 10 years.
Recruitment	· EPIC-Norfolk: The EPIC-Norfolk study recruited middle-aged individuals from the general population of Norfolk, a county in Eastern England, who attended the baseline assessment between 1993–1998.<br>· Other studies: All other studies described their recruitment methods in their respective studies, which are cited in the References section
Ethics oversight	All contributing cohorts obtained ethical approval from their intuitional ethics review boards. The contributing cohorts include UK Biobank, GIANT consortium, deCODE study, Fenland study, AGES Reykjavik study, INTERVAL study, CARDIoGRAMplusC4D, GIGASTROKE, and MAGIC consortium. For individual-level data, the study was approved by the UK Biobank (application number: 27449) and the Norfolk Research Ethics Committee (no. 05/ Q0101/191), and all participants

gave their informed written consent. For human adipose-derived mesenchymal stem cells used in LipocyteProfiler, each participant gave written informed consent before inclusion and the study protocol was approved by the ethics committee of the Technical University of Munich (Study No 5716/13) and the Broad Institute of MIT and Harvard (IRB number: ORSP-1613).

Note that full information on the approval of the study protocol must also be provided in the manuscript.

Field-specific reporting

Please select the one below that is the best fit for your research. If you are not sure, read the appropriate sections before making your selection.

☒ Life sciences ☐ Behavioural & social sciences ☐ Ecological, evolutionary & environmental sciences

For a reference copy of the document with all sections, see nature.com/documents/nr-reporting-summary-flat.pdf

Life sciences study design

All studies must disclose on these points even when the disclosure is negative.

Sample size	The sample size of each dataset is provided in Supplementary Table 1.
Data exclusions	Plasma proteins whose cis-pQTLs were not available in the largest proteomic GWAS study were excluded from Mendelian randomization (MR) analyses evaluating the effect of obesity on cardiometabolic outcomes.
Replication	Apart from the primary discovery MR analysis with data from deCODE, we performed another set of MR using independent cohorts: the UK Biobank, the Fenland study, and the ARIC study.
Randomization	Not applicable since this is not an interventional study.
Blinding	Not applicable since this is not an interventional study.

Reporting for specific materials, systems and methods

We require information from authors about some types of materials, experimental systems and methods used in many studies. Here, indicate whether each material, system or method listed is relevant to your study. If you are not sure if a list item applies to your research, read the appropriate section before selecting a response.

Materials & experimental systems

n/a	Involved in the study	
☒	☐	Antibodies
☐	☒	Eukaryotic cell lines
☒	☐	Palaeontology and archaeology
☒	☐	Animals and other organisms
☒	☐	Clinical data
☒	☐	Dual use research of concern
☒	☐	Plants

Methods

n/a	Involved in the study	
☒	☐	ChIP-seq
☒	☐	Flow cytometry
☒	☐	MRI-based neuroimaging

Eukaryotic cell lines

Policy information about cell lines and Sex and Gender in Research

Cell line source(s)	Human adipose-derived mesenchymal stem cells were obtained from the Munich Obesity BioBank (MOBB).
Authentication	The cells were not authenticated.
Mycoplasma contamination	The cells were negative for mycoplasma.
Commonly misidentified lines (See ICLAC register)	Not used in the study.
